# Effect of a System-Oriented Intervention on Compliance Problems in Schizophrenia: A Pragmatic Controlled Trial

**DOI:** 10.1155/2014/789403

**Published:** 2014-06-02

**Authors:** Hanne Skarsholm, Henrik Stoevring, Bent Nielsen

**Affiliations:** ^1^The Psychiatry in the Region of Southern Denmark, Søndre, Sdr. Boulevard 29, 5000 Odense C, Denmark; ^2^Department of Public Health, Aarhus University, 8000 Aarhus C, Denmark; ^3^Department of Psychiatry, The Psychiatry in the Region of Southern Denmark, 5000 Odense, Denmark

## Abstract

*Background*. Numerous studies have been conducted with a view to developing strategies for improvement of medical compliance in patients with schizophrenia. All of the studies conducted so far have had an individual approach to compliance based on the assumption that noncompliance is determined individually due to inappropriate behavior in the patient. We conducted a pragmatic controlled trial with a system-oriented approach, to provide a new perspective on compliance and test the efficacy of a multifactorial intervention at the system level in a routine clinical setting, an approach that has not previously been used for the improvement of compliance. *Methods*. 30 patients were allocated to the system-oriented therapy and 40 patients were allocated to the reference intervention, which consisted of individually based compliance therapy. The follow-up period was six months. Primary endpoint was improvement in compliance, measured by improvement in a compliance scale specifically developed for the project. *Results*. When accounting for missing values with a multiple imputation approach, we found a tendency toward a difference in both the compliance scale and PANSS favoring the system-oriented therapy, although it did not reach statistical significance. A significant difference in incidence of adverse events and time to first readmission was found. Attrition rates were significantly higher in the reference group and nonsignificant among individuals with lower compliance, which may have diluted effect estimates. This was reflected by significant differences found in an analysis based on a last observation carried forward approach. *Conclusion*. This study suggests that compliance problems are better solved by a multifactorial intervention at the system level than at the individual level.

## 1. Background

Schizophrenia is a severe mental illness [1], for which the cornerstone of treatment is antipsychotic medication [2]. Compliance with the medications is poor, as only approximately 50% of the prescribed medication is consumed [3], even though a compromised compliance has extensive clinical and economic consequences [4, 5]. Consequently, numerous studies have been conducted to develop strategies for the improvement of compliance in schizophrenia [6–18]. These studies have all had an individual approach to compliance based on the assumption that noncompliance is individually determined and due to inappropriate behavior of the patient. Alternatively noncompliance could be regarded as an “incident” caused by a “system failure” [19], that is, a product of failed processes within the organization. From this perspective, key elements in improving compliance would be to establish monitoring to minimize errors, build barriers that prevent errors, and perform careful analysis when errors are made in order to identify causes and prevent recurrence. This approach uses principles of clinical risk management and quality assurance principles [20].

Based on a system-oriented approach, we will in this study bring a new perspective on compliance and test the efficacy of a multifactorial intervention at the system level in comparison with a more traditional individual based intervention with respect to improving compliance in patients with schizophrenia, which as far as we know has not previously been done.

The study is a pragmatic controlled trial in the sense that interventions were delivered in a routine general adult psychiatric setting so as to maximize generalisability of its results.

## 2. Methods

### 2.1. Study Setting and Design

Two open psychiatric sections at the Department of Psychiatry, Odense, Denmark, participated in the study with their community mental health teams assigned to the respective sections. Each section and assigned team received patients from geographically well-defined areas in Odense and there was no significant difference in the patient population or staff composition in observed characteristics. The patients were allocated to one of the two participating psychiatric sections according to their home address. In one section, the system-oriented intervention was implemented, and the individual-oriented approach was in the other section.

**Figure 1:**
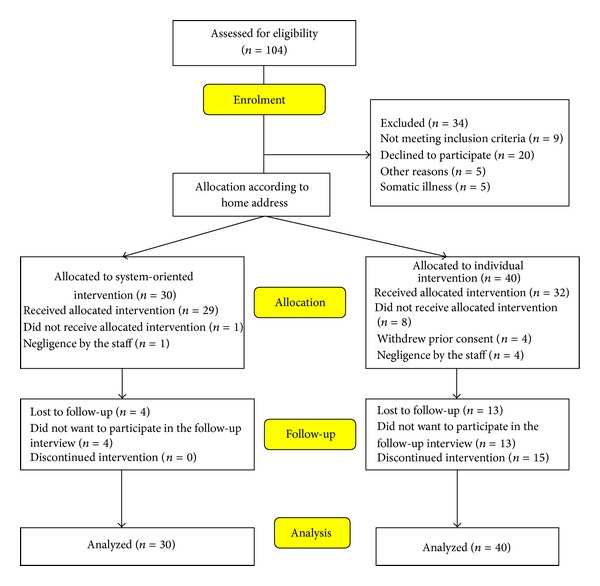
Flow chart.

The flow diagram shows the study design schematically (Figure 1).

Patients, who fulfilled the inclusion criteria, were invited to participate in the study within a few days after admission. If they accepted, informed written consent was obtained and an index interview conducted before the intervention was started. The follow-up period ended with an interview after six months.

Medical records were collected to obtain information on readmissions and bed days from baseline and in the subsequent 12 months.

The two intervention groups were considered to represent a reference intervention and an experimental intervention, respectively. We chose to use a reference group rather than a control group with treatment as usual, since compliance problems tend to disappear when focusing on them, regardless of the nature of the intervention [21].

### 2.2. Study Population

Criteria for including patients admitted in the study period were ICD-10 diagnosis of schizophrenia or schizoaffective disorder; age of 23–70 years; Danish ethnicity; being supposed to be followed up by the community mental health teams; and not being subject to legal action.

104 patients were assessed to be included of which 34 were excluded; see Figure 1. The intervention groups thus consisted of 30 patients in the system-oriented intervention group and 40 patients in the individual group.

The study was conducted in accordance with the Declaration of Helsinki and approved by The Ethics Committee, Region of Southern Denmark, Denmark.

### 2.3. Interventions

#### 2.3.1. The System-Oriented Intervention

The system-oriented intervention will be briefly introduced here—for further details please see Skarsholm and Nielsen [22].

The system-oriented intervention was based on five standards (qualitative), which intended to improve medical compliance. The five standards dealt with the following topics: information, compliance, reminders, medication reconciliation, and clinical guidelines for treatment with antipsychotic medications.

*(1) Information*. Standard concerning information on antipsychotic drug treatment.

The standard contained a brochure on antipsychotic drug treatment and a questionnaire as a basis for conversation between the patient and his or her primary care provider about the patient's experience of information.

*(2) Compliance*. Standard concerning focus on identifying and solving compliance problems.

The standard was designed to ensure a regular focus on compliance issues in conversations between patient and nurse by using a screening form for identification of compliance problems. Identified obstacles should be sought removed.

*(3) Reminders*. Standard concerning reminder systems.

The content of this standard was a “reminder box.” It contained among f x medicine cards, medicine dosage boxes, electronic alarm systems for reminding of medication intake, and reminder of prescription renewal. There were no restrictions on the content of the box, and it was allowed to add relevant reminders during the project period.

*(4) Medication Reconciliation*. Standard concerning medication reconciliation at transfer.

This standard focused on avoidance of potential medication errors when transferring between different sectors. The concept of medication reconciliation involves control of medication in any relocation including discharge.

*(5) Clinical Guidelines for Treatment with Antipsychotic Medications*. The guidelines were developed by psychiatrists and clinical pharmacologists affiliated to Odense University Hospital after the extensive literature studies. The clinical guidelines should provide the standard for good clinical practice in antipsychotic drug therapy.

The performance of the standards was recorded in monitoring schemes and evaluated at audits twice a year with subsequent feedback to the clinical team.

For patients readmitted with compliance problems a special in-depth audit, so-called “aggregate root cause analysis” (http://www.patientsafety.gov/), was performed, which means that multiple occurrences of the same character accrued over a given time were analyzed simultaneously in order to identify causes for noncompliance and give feedback to the care providers. This method is ideal where there are likely frequent occurrences within a given period, especially when these occurrences look like each other, meaning that you can identify common trends in the causes you find for non-compliance in the analyses.

Prior to the inclusion of patients the involved staff participated in a four-day course consisting of training in psychopharmacology, psychopathology, quality and risk management, root cause analysis, and compliance promotion.

#### 2.3.2. The Individual Intervention

The individual intervention consisted of compliance therapy, which was a manual based short-term therapy, based on an individual, cognitive-behavioral therapeutic approach to improvement of compliance in people with schizophrenia. It was built on the principles of “motivational interviewing” [23] and “concordance skills” [24]. It consisted of 6 sessions and 3 booster sessions each of 30–45 minutes' length.

The therapy consisted of 6 areas: (1) assessments of current medication, attitudes towards medication, and adverse events, (2) solving problems related to medication, (3) reviewing former positive and negative experiences with different treatment strategies, (4) study of ambivalence, (5) concerns and expectations in relation to medicine, and (6) the future, including relapse prevention and warning signals.

In all phases the motivational interviewing style was used, which included clarifying the discrepancy between the patient's desires and behavior in relation to medicine. The aim of the therapy was to achieve joint decision making about medicines, since a central tenet of the therapy was that a joint decision will increase compliance.

Prior to the inclusion of patients a four-day course was held for the staff involved in the individual intervention group, consisting of education in psychopharmacology and psychopathology and training in “motivational interviewing.” The teacher in “motivational interviewing” was trained by the founder of the therapy and was responsible for the ongoing supervision during the project.

Both interventions were intended to be initiated in the ward and continued in the community mental health teams.

### 2.4. Objectives and Outcomes

The primary endpoint was improvement on a compliance scale specifically developed for the project and consisting of 4 items: (1) compliance self-assessment by the patient, (2) Drug Attitude Inventory (DAI-10) [24], (3) appointment keeping with the common mental health system, and (4) PANSS-item G12 concerning judgment about illness and need for treatment [25]. All items are proven methods to assess compliance [26–32], but no method is considered to be the gold standard. A combination of measures maximizes accuracy [3].

Validation of the scale resulted in Crohnbach's alpha coefficient of 0.81. Loevinger's coefficient, *H*, was 0.61, 0.64, 0.52, and 0.62 of items 1, 2, 3, and 4, respectively. Possible scores on the compliance scale ranged from 0 to 8 points.

The secondary endpoints were improvement in a number of recognized clinical scales, as an indirect measure of improved compliance: Positive and Negative Syndrome Scale's (PANSS) remission criteria [25], Global Assessment of Functioning Scale (GAF) [33], and Subjective Well-Being on Neuroleptic Treatment Scale (SWN) [34].

The UKU side effects rating scale (UKU) [35] was also used as the presence of side effects indicates actual consumption of the medication.

The tertiary endpoints were time from discharge to first rehospitalization and bed day consumption within 12 months.

### 2.5. Statistical Analysis

Prior to enrolment of patients a power calculation was undertaken. We assumed an improvement in compliance score of at least 1.00 points on average. We also assumed that there would be a baseline standard deviation of compliance points of maximum 1.00. Under these conditions a minimum of 40 participants in the project was needed in order to achieve a power of 80% at a significance level of 5%.

For comparison of unpaired, numeric, nonnormally distributed data, the Mann-Whitney test was applied. For comparison of unpaired, numeric, normally distributed data, the *t*-test was used. To provide confidence intervals for estimated differences of nonnormally distributed data, robust variance estimates were used. For comparison of categorical data, Fisher's exact test was applied. The two-sided significance level was set to 5%.

All participants were primarily analyzed by the intention-to-treat principle with the method of multiple imputation (MI) and alternatively with the last-observation-carried-forward (LOCF) method.

To account for missing data by MI, we identified the baseline variables that were best predictors of whether a subject completed follow-up and used these in a multiple imputation to predict missing values of the outcomes. Based on the literature we assumed that low GAF score, age, duration of illness, compliance at baseline, and abuse status were both related to whether a patient would fail to complete follow-up and what the missing values would have been had they not been missing. 10 sets of multiply imputed data were generated and analyzed.

All analyses were conducted in Stata 10.

## 3. Results

### 3.1. Characteristics and Disposition of Patients

Table 1 shows the baseline comparison of patients for a variety of demographic and clinical conditions. There were no significant differences between the two groups at baseline.

### 3.2. Discontinuation and the Intervention Process

There was a significantly greater proportion of the individual compared to the system-oriented group that did not complete follow-up (15/40 versus 4/30, *P* value 0.017). In the individual intervention group, each participant received on average 3 sessions (SD 2.1) and 0 booster sessions (SD 0.7).

The system-oriented intervention had a fulfillment of the standards of 43% in the hospital. In the common mental health teams, the standards were met by 57%. The compliance with the standards was highest at the beginning of the study (attention bias).

Four aggregate root cause analyses were performed during the intervention period. They concurrently revealed instances of nonuse of standards and failure to use information and knowledge about the patient's condition.

### 3.3. Effect of the Intervention on the Compliance Scale (Primary Endpoint)

Analysis of data with MI yielded a coefficient of 0.476 (SE 0.362 and CI −0.247–1.120); that is, there was a positive effect on compliance associated with being in the system-oriented group rather than the individual group after adjustment for compliance at baseline, abuse status, and GAF. Abuse status and GAF are known from the literature to affect compliance and were hence controlled for to improve precision. The result was not significant, *P* value 0.193 (Table 2).

### 3.4. Effect of the Intervention on the Rating Scales (Secondary Endpoints)

*Remission*. There was no significant difference at follow-up.

*PANSS*. Regression using MI leads to a regression coefficient of PANSS at follow-up in the intervention group when adjusted for PANSS at baseline of −4.478 (CI −9.259–0.403), *P* value 0.072.

LOCF revealed a significantly lower PANSS score on the remission items in the system-oriented group at follow-up, on average a score of 22 compared to 26 in the individual group, *P* value 0.036. When the individual's baseline value was included, *P* value of the differences was 0.001 by *t*-tests in favor of the system-oriented group. Estimate of the difference between the two groups by regression was 4.93 (CI −7.835 to −2.015).

*GAF and SWN*. No significant difference could be identified (MI and LOCF).

*UKU*. Analysis based on MI leads to an OR of 4.218 (CI 1.427 to 12.470) comparing the system- to the individual-oriented intervention. Based on LOCF 17 (43%) in the individual group and 21 (70%) in the system-oriented group were assessed as having adverse events, *P* value 0.030.

### 3.5. Data on Bed Days (Tertiary Endpoints)

**Figure 2:**
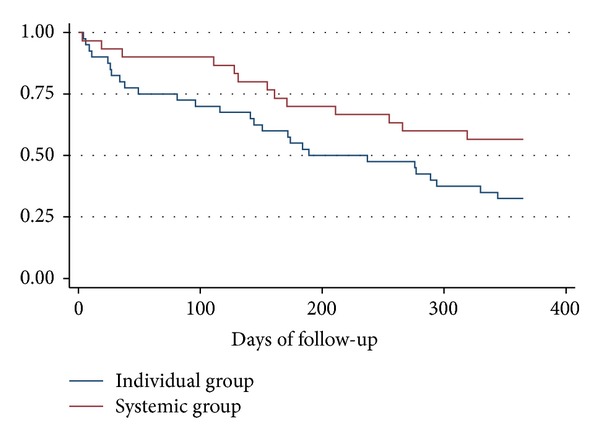
Kaplan-Meier survival proportion.

These data were collected from medical records of all the 70 patients.  Figure 2 shows the proportion of project participants divided by intervention group, who have not been readmitted at a given point of time.

The system-oriented group had significantly longer time to readmission, *P* value 0.049.

There was no significant difference in the duration of the index hospitalization in the two groups or the average bed day consumption throughout the 12 months.

### 3.6. Nonparticipants

There were 16 patients who met the criteria for enrolment but who did not want to participate in the project (Figure 1). These were significantly older than participants.

## 4. Discussion

When accounting for missing values with a multiple imputation approach, we found a tendency toward a difference in both the compliance scale and PANSS favoring the system-oriented therapy, although it did not reach statistical significance. A significant difference in incidence of adverse events and time to first readmission was found. Attrition rates were significantly higher in the reference group and nonsignificantly among individuals with lower compliance, which may have diluted effect estimates. This was reflected by significant differences found in an analysis based on a last observation carried forward approach.

More results were not significant using MI because MI takes into account the uncertainty of the missing data. MI thus best reflects the actual uncertainty of results.

The patients were allocated to one of the two participating psychiatric sections according to their home address in order to avoid confounding by indication. Still the combination of an unblinded treatment in a pragmatic trial with patient self-assessment holds potential for bias. To protect against this, pragmatic trials may benefit from including objective outcome measures in addition to subjective measures [36]. This was the case in our study, where time to readmission was an objective outcome, which furthermore was available for all at end of follow-up and showed results in the same direction as when analyzing subjective outcome measures.

The systemic intervention had an effect on the compliance level and was better tolerated than the individual-oriented intervention since no patients in the systemic intervention group withdrew from the project during the intervention period.

It was expected that during the project period there would be an increasing degree of compliance with the standards, but this was not achieved. The fulfillment was highest in the first period, which may be regarded as an attention bias (Hawthorne effect).

It was difficult to optimize the quality of daily routines as indicated by the four root cause analyses largely having similar conclusions. More focus on “knowledge sharing” was needed—focus on the factors influencing uptake and retention of new knowledge among health care staff [37].

No significant effect of the individual intervention was obtained. The individual intervention was poorly carried out. It was supposed to consist of 6 sessions and 3 booster sessions; yet the patients received on average 3 sessions and 0 booster sessions. Furthermore evaluation by the supervisor gave the impression of inadequate acquisition of the method (motivational interviewing) of those who were supposed to carry out the therapy. The supervisor estimated that training would have to be doubled for it to become adequate. After completion of the investigation, contact with W. Miller, who founded the therapy, clarified that experience has shown large variations in the amount of training needed to achieve sufficient skills to perform adequately (private communications). In the literature, divergent effects of compliance therapy and adherence therapy have been reported [8, 10, 12, 38, 39].

A study from 2010 [18] found no effect of adherence therapy on either clinical symptoms as measured by PANSS or medical adherence. The study was probably affected by selection bias, since only 26 of the 130 possible individuals accepted participation.

Consequently, the effect of compliance therapy and adherence therapy must still be regarded as uncertain, especially after the recent studies [40].

With 70 participants, we achieved more than the 40 participants assumed to be sufficient according to the power calculation. However, the actual size of the observed effect and standard deviations demanded a bigger amount of participants to achieve significant results. Usually pragmatic trials require large sample sizes to detect small treatment effects in a heterogeneous population [36].

The project was designed as a pragmatic controlled study, which gives a realistic impression of the effect of intervention, as it would be when carried out in a routine clinical setting, because such trials are performed in the context of usual care and use broad eligibility criteria [41]. It seeks to address the overall effectiveness of a given therapy [36, 42] and is therefore useful when considering the benefits of implementing a new treatment, even if this way of conducting a study may sacrifice intern validity to achieve generalisability [36, 43].

The patients were allocated to hospital section, and thus intervention type, from their residential address in order to avoid selection bias, specifically “confounding by indication.”

The literature recommends cluster randomization for testing system-level interventions since patient randomization may be vulnerable to contamination. Patients in the reference group may be influenced by the system-level changes [44]. Therefore, the two interventions were applied at two different sections.

This design allowed us to implement and test a system-based intervention, which has not been previously done in this vulnerable group of patients.

## 5. Conclusion

This study suggests that compliance problems are best solved by a multifactorial intervention on the system level. Yet the point that the system-oriented intervention was superior to the individual intervention can be questioned; since the patients only received small amounts of this intervention, it still can serve as a reference intervention recording to the fact that compliance problems tend to disappear when focusing on them (attention bias).

The system-oriented intervention is likely suitable to be applied to compliance problems in other groups of vulnerable patients with chronic diseases.

This study may be considered a first step in investigating the potential effect of system-based interventions on improving patient compliance—a perspective which in our view has not received sufficient attention in the past. Although not all the estimated effects reached significance, they all favored the systemic intervention and they should therefore serve as the basis of larger and more definitive studies of this kind.

## Figures and Tables

**Table 1:** Baseline demographic and clinical characteristics.

	Individual intervention, *N* (%) Mean (SD) Median [10; 90% percentile]	Systemic intervention, *N* (%) Mean (SD) Median [10; 90% percentile]	*P*
Number	40	30	
Sex (%)			
Male	17 (42.5)	14 (46.7)	
Female	23 (57.5)	16 (53.3)	0.810
Age, yr (SD)	43.4 (10.6)	40.1 (10.0)	0.195
High school (%)	19 (47.5)	12 (40.0)	0.190
Education (%)	18 (45)	15 (50)	0.810
Livelihood (%)			
Employed	2 (5.0)	1 (3.3)	
Student	1 (2.5)	0 (0.0)	
Early retirement (financed by the state)	35 (87.5)	26 (86.7)	
Cash benefits	1 (2.5)	2 (6.7)	
Others	1 (2.5)	1 (3.3)	0.949
Crime (%)			
Arrested	10 (25.0)	9 (31.0)	0.597
New diagnosed (%)	2 (5.0)	2 (6.7)	1.000
Number of admissions	8.5 [2; 31.5]	10 [1; 50]	0.384
Duration of illness, yr	19 [4; 30]	11 [5; 32]	0.301
Abuse			
Alcohol and/or drugs	10 (25.6)	14 (46.7)	0.080
Alcohol (+ drugs)	8 (20.5)	13 (43.3)	0.064
Community mental health team	26 (65.0)	21 (0.7)	0.798
Psychosocial functioning (GAF)	33 [25; 40]	33 [25; 45]	0.727
Taking medication	34 (85.0)	28 (93.3)	0.425
Medications			
First-generation drug	9 (22.5)	8 (26.7)	0.781
Second-generation drug	31 (77.5)	26 (86.7)	0.371
Clozapine	10 (25.0)	10 (33.3)	0.594
Depot antipsychotics	10 (25.0)	11 (36.7)	0.307
Polypharmacy	10 (25.0)	10 (33.3)	0.594
Side effects			
Physician assessment	25 (62.5)	20 (66.7)	0.804

Missing values: crime 1, number of admissions 1, duration of illness 30, abuse 1, SWN 4, PANSS, all scores 1, remission 1, GAF 2, and side effects 7.

SWN: Subjective Well-Being under Neuroleptic Treatment. PANSS: Positive and Negative Symptom Scale. GAF: Global Assessment of Functioning Scale. Remission: score max 3 on all of the PANSS remission items.

**Table:** (a) Difference in compliance score from baseline to follow-up, LOCF

	Difference, compliance score mean (CI)	*P* value
The individual therapy, *N* = 40	0.400 (−0.174–0.974)	>0.05
The system-oriented therapy, *N* = 30	1.103 (0.434–1.733)	<0.05

**Table:** (b) Test on difference between intervention groups

	Coefficient	SE	CI	*P* value
Regression, MI	0.476	0.362	−0.247–1.120	0.193
Regression, LOCF	0.724	0.338	0.050–1.398	0.036
